# Development of automated postoperative enteral nutrition: restricting feeding site inflow to match peristaltic outflow

**DOI:** 10.1186/s13022-015-0022-1

**Published:** 2015-12-11

**Authors:** Gerald Moss

**Affiliations:** Rensselaer Polytechnic Institute, Troy, NY USA; 1 Reynal Road, 10605 White Plains, NY USA

**Keywords:** Automatic feeding control, Postoperative enteral nutrition, Peristalsis monitoring, Wound healing, Immune competence

## Abstract

**Background:**

Surgical stress accelerates postoperative metabolism, while simultaneously compromising gut activity. The dysfunction may be worsened by early feeding. These patients are not expected to fully meet their optimum metabolic requirements using current nutritional regimens. For optimum postoperative enteral nutrition, we must automatically match the patients’ feeding site inflows to their impaired peristaltic outflows. An essential adjunct is virtually complete exclusion of swallowed air. The small diameter post-pyloric duodenum is an efficient site for both aspiration and feeding.

**Method:**

A multi-lumen feeding-decompression tube is utilized to feed an “elemental diet” (1 kcal/ml) at the rate of 3000–5000 ml/day immediately postoperatively. Outflow from the feeding site, relative to inflow, is monitored. Excess aspirate is discarded automatically, so that the re-fed inflow exactly matches the peristaltic outflow. The aspirate (digestive secretions, feedings, and swallowed air) is degassed. The aspirate is then directed by one-way valves alternately, every 30 s, into a pair of 30 ml collection chambers for re-feeding. Simultaneously, the previously collected aspirate (less discarded volume, if required) is delivered by gravity into the slightly more distal duodenum.

**Results:**

Within 2 h, the return of the entire aspirate volume is tolerated. The patients’ increased metabolic demands are met early on the initial day of surgery. They achieve positive protein balance, with documented enhanced healing (experimentally) and immune globulin synthesis (clinically). Breakfast is tolerated the morning following operation, with discharge soon thereafter. X-ray motility and nutrient absorption studies document the more rapid return of clinically normal peristalsis.

**Conclusion:**

Automatically keeping the proximal duodenum and stomach continuously decompressed, while simultaneously re-feeding tolerated degassed duodenal aspirate, leads to more rapid return of clinically adequate G-I function postoperatively. In addition to maximizing immediately postoperative nourishment, the secretory globulins are salvaged, and spontaneously delivered into the colon, where they provide natural antimicrobial protection.

## Background

Surgical stress increases a patient’s metabolic activity, while simultaneously impairing postoperative G-I function. As a rule, we expect these patients will be unable to safely and spontaneously optimally meet their increased metabolic requirements. Peristaltic activity will be impaired, and the degree of dysfunction may be worsened by “overfeeding”.

The benefits of early enteral nutrition are indisputable, yet relative starvation invariably follows major abdominal surgery. Postoperative paralytic ileus and the fear of aggravating this complication lead to under-nutrition. The hyper-metabolic response to trauma in the face of inadequate nourishment accelerates proteolysis and retards protein synthesis. Healing is slowed, vulnerability to infection increases, and patients weaken. They also suffer the complications and subjective discomforts of sluggish gut function. There are major economic implications.

At the 1963 Annual Meeting of the American College of Surgeons, we reported the first clinical achievement of positive protein balance by enteral feeding within hours of bowel resection [1, 2]. Our multi-lumen nasal catheter removed swallowed air by efficiently aspirating the esophagus.

**Fig. 1:**
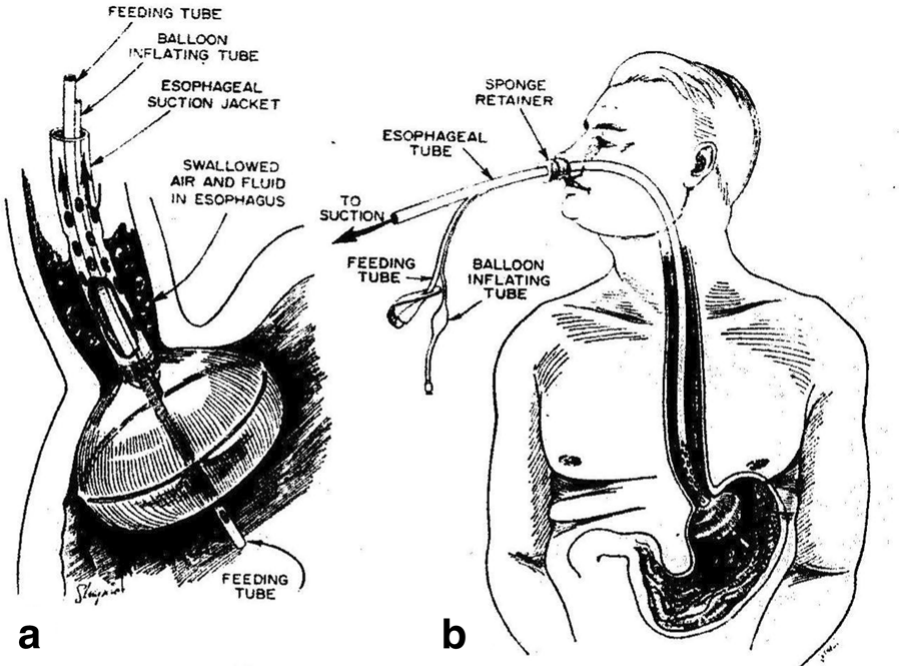
Naso-esophageal decompression—gastric feeding tube. 1963 American College of Surgeons Surgical Forum. Refs. [1, 2]

Gastric tube feeding and frequent monitoring of emptying were performed manually (Fig. 1).

This result was anticipated by Wangensteen and his Minneapolis team in the 1930’s. They showed that aerophagia was a key factor in the development of bowel dysfunction [3]. Even the usually disastrous consequences of complete distal small bowel obstruction could be avoided by exclusion of swallowed air from the intestinal tract.

Wangensteen transected and over-sewed the canine terminal ileum. Saliva and swallowed air were vented via a cervical esophagostomy, and nutritional support was limited to subcutaneous Ringer’s solution. The dogs survived for up to 2 months, despite total bowel obstruction. They lived an average of 1 month before dying of starvation, and without developing intestinal distention or other signs of G-I dysfunction. We had unknowingly incorporated Wangensteen’s over 20-year-old insights into our earliest approach to postoperative enteral nutrition.

Our clinical regimen could not be replicated readily in the usual setting. The patients raised and immediately swallowed copious quantities of bronchial secretions. Herculean nursing efforts were required to keep the esophageal suction channel and aspiration orifices patent. This labor-intensive attention was available only at facilities such as our NIH funded Clinical Research Center at Albany Medical Center Hospital.

**Fig. 2:**
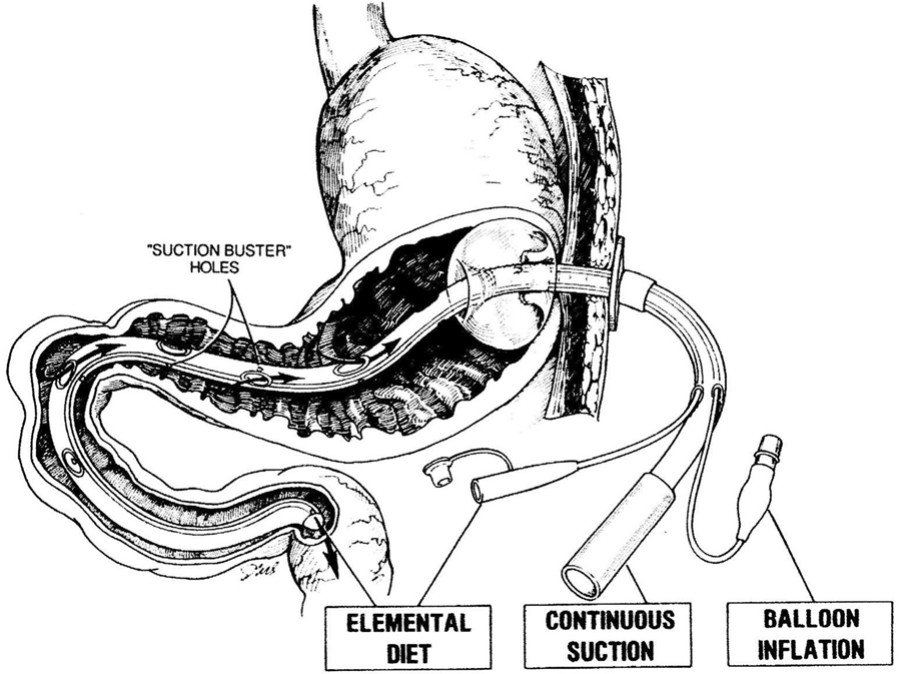
Current feeding/decompression gastrostomy tube (1987). Ref. [4]

**Fig. 3:**
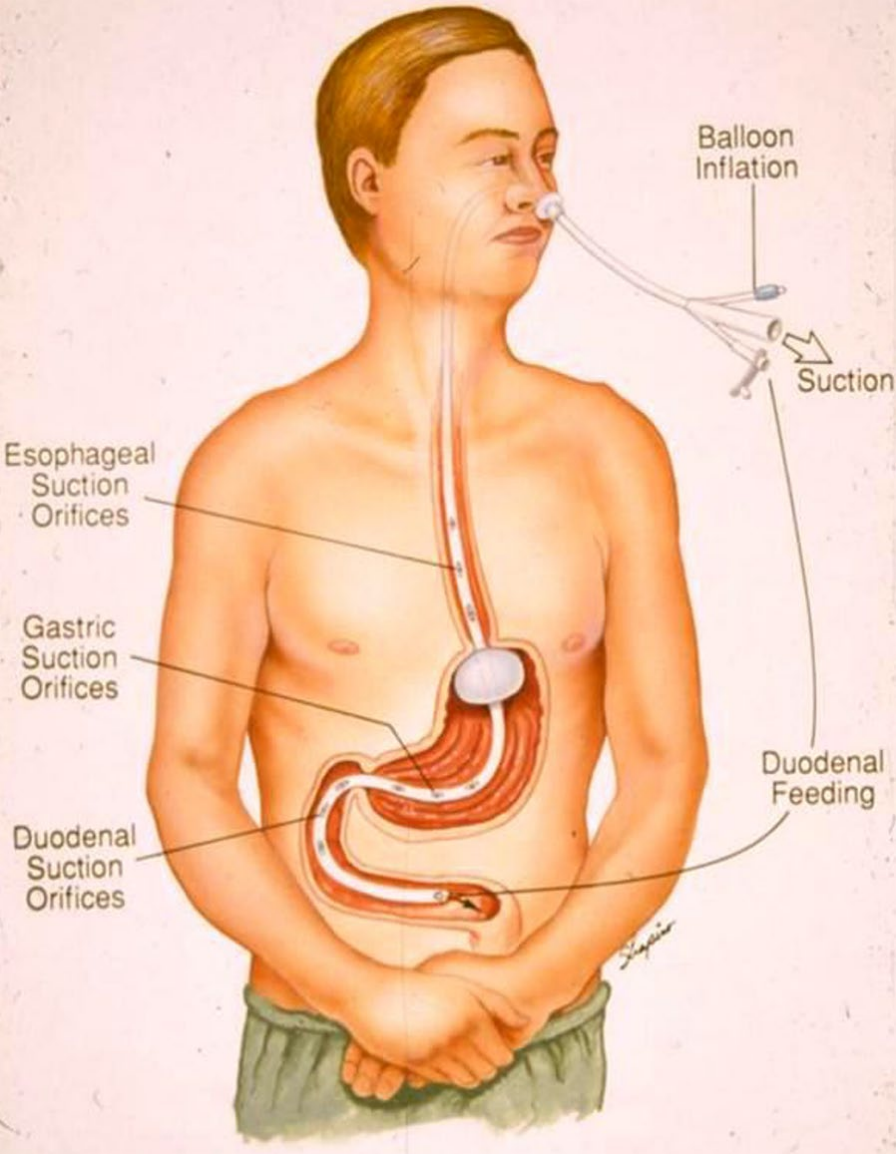
Current nasal feeding/decompression feeding tube (1987). ref. [5]

The catheters were modified to make the regimen more widely acceptable [4] (Moss Tubes, Inc., West Sand Lake, NY) (Figs. 2, 3). The aspiration site for effective air interception and removal was relocated beyond the pylorus, and feedings were delivered into the slightly more distal duodenum. This allowed for dilution of the swallowed bronchial secretions, slowing occlusion of the aspiration channel.

Aspiration orifices also were located within the stomach. This necessary adjunct prevented gastric distention, which alone could have disrupted G-I function. Residual liquid and air that escaped gastric aspiration were intercepted more efficiently within the simultaneously aspirated, smaller diameter duodenum.

Any inflow that exceeded the patients’ duodenal outflow freely refluxed a short distance into the aspiration zone, to be removed while still within the duodenum. The geometry and physiology of the small diameter esophagus and duodenum made them over twelve times more effective aspiration sites than the stomach, facilitating interception and removal of essentially all swallowed air [5].

Continued reports of successful postoperative “enteral hyper-alimentation” with reduction of hospital stay were encouraging [6]. Despite a hyper-metabolic state requiring about 3000–5000 kcal/day of balanced nutrition for satisfaction, positive protein balance was reported consistently within hours of major surgery.

**Fig. 4:**
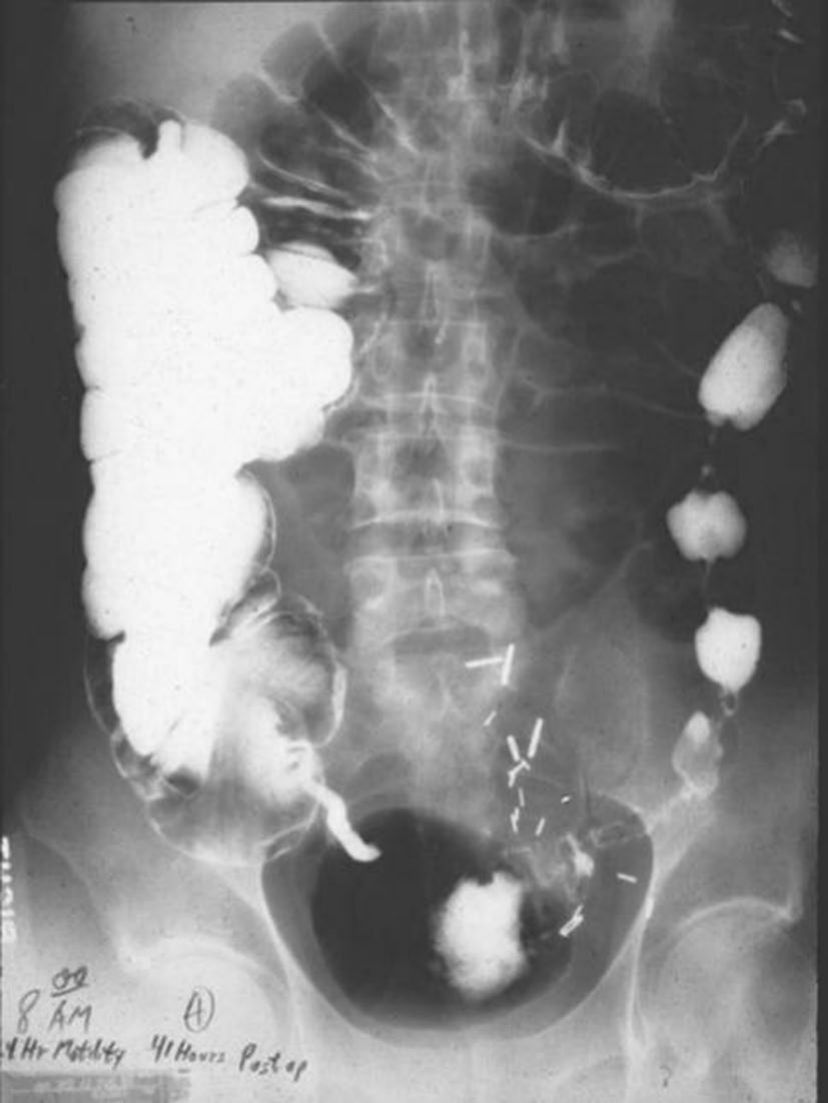
24 h motility study—41 h after sigmoid resection (1977). Discharge 43 h after sigmoid resection for carcinoma (prior to laparoscopy). Clinically normal peristalsis in the descending colon and anastomosis. Ref. [4]

**Fig. 5:**
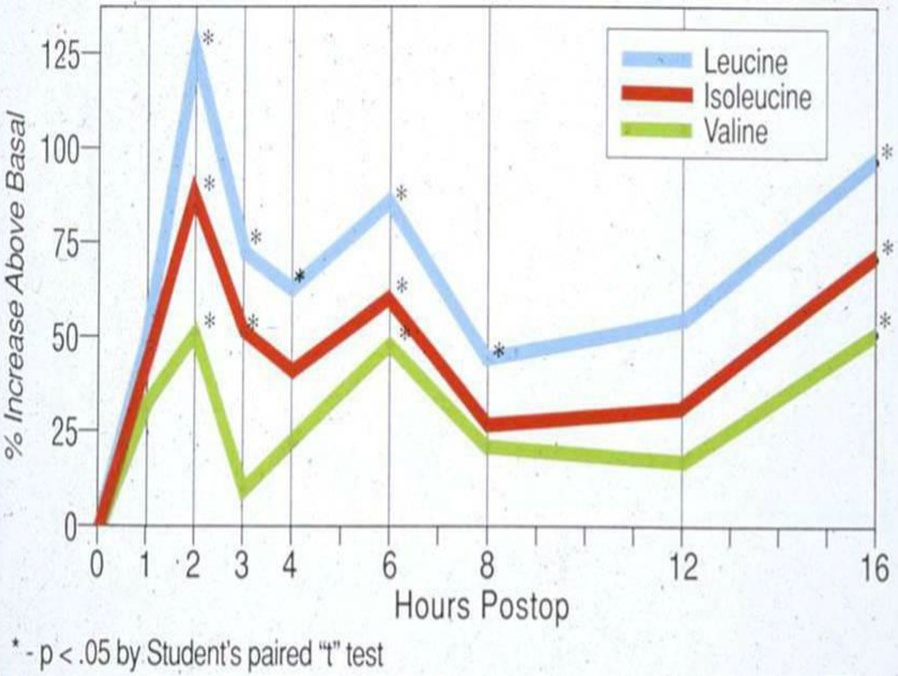
Serum aa’s postcholecystectomy. “Enteral hyperalimentation” + duodenal decompression. Refs. [7–9]

**Fig. 6:**
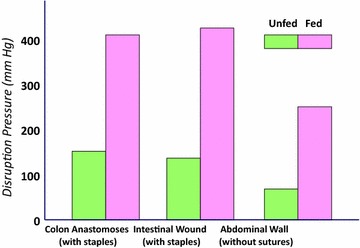
Canine anastomatic wound strength. Wound bursting pressures on day #4, fed versus unfed. Refs. [10–12]

**Fig. 7:**
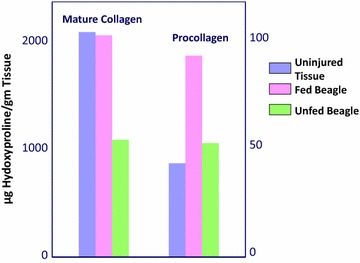
Canine bowel wound collagen and procollagen. Anastomotic collagen day #4, fed versus unfed. Refs. [10–12]

**Fig. 8:**
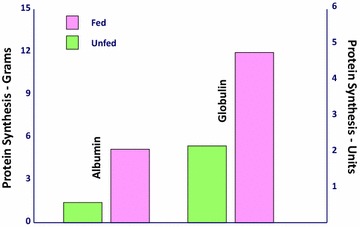
Canine plasma protein synthesis day #1. Bowel resection day #1, fed versus unfed. Refs. [11–13]

**Fig. 9:**
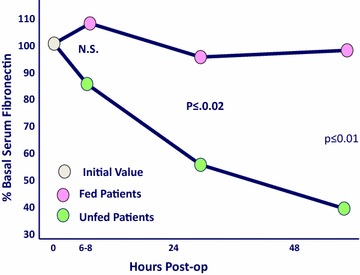
Postcholecystectomy fibronectin. Postop fibronectin—fed versus unfed. Refs. [11–13]

**Fig. 10:**
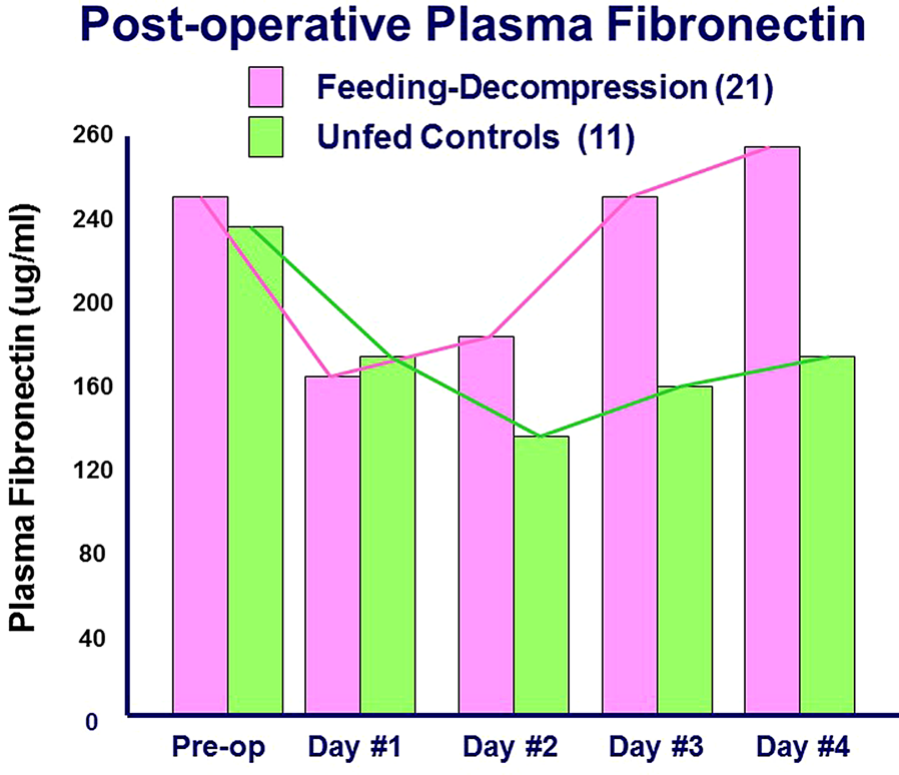
Radical resections postoperative plasma fibronectin. Roswell Park Cancer Institute, fed versus unfed. Ref. [13]

Within 2 h of operation, no elemental diet was detectable in the aspirate. All the feedings had been propelled downstream to be completely digested. Absorption and X-ray motility studies confirmed the rapid recovery of G-I function [7–9] (Figs. 4, 5). Healing was accelerated, and antibody production was increased [10–12] (Figs. 6, 7, 8, 9). In controlled clinical studies at Roswell Park Cancer Institute (Buffalo, NY) with our regimen, “enteral hyperalimentation” resulted in significantly elevated early levels of fibronectin, indicative of accelerated healing and immune competence [13] (Fig 10). Adequate enteral nutrition could be absorbed soon after major surgery to meet the patients’ increased metabolic demands. The challenge still remained to more practically exploit this nutritional potential.

In addition to still requiring increased nursing attention, albeit less than with our previous approach, this regimen caused an obligatory loss of about 4000 ml of digestive secretions, analogous to a duodenal fistula. This was met by massive intravenous fluid and electrolyte replacement, guided by laboratory studies. The discarded juices also contained large amounts of secretory globulins, specific to control the patients’ own colonic organisms.

In 2007, we reported an approach to automatically re-feed the aspirate after thoraco-abdominal esophageal resection for carcinoma [14]. Jejunal aspirate was diverted for 30 s into a 30 ml chamber. Aspiration was interrupted for the next 30 s, while the aspirate was re-fed via the delivery channel.

**Fig. 11:**
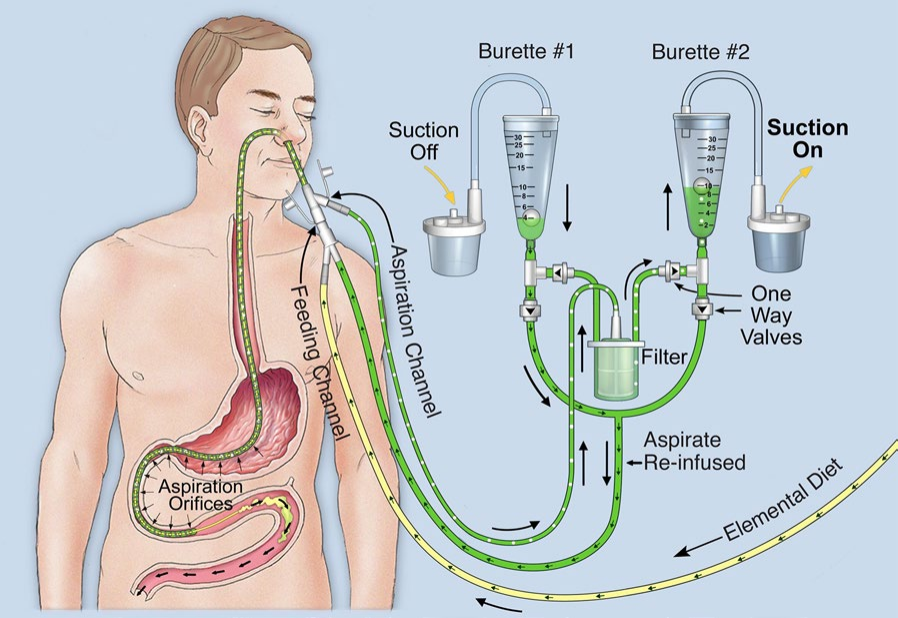
Automatic monitoring and feeding control

This system was modified to make it universally applicable after surgery (Fig. 11). The stomach and proximal duodenum were aspirated continuously, to eliminate the opportunity for air to be propelled beyond the aspiration zone while suction was interrupted. The aspirate flowed alternately, at 30 s intervals, into one of two 30 ml collection chambers. Simultaneously, the previously collected aspirate was re-fed into the more distal duodenum. The need to discard excessive aspirate ceased within 1–2 h postoperatively, as the minimally required peristaltic activity had been restored. Only about 100–200 ml of excess aspirate was discarded the first day, and decreased thereafter.

The feedings had to remain soluble after contact with gastric and/or intestinal fluids. Protein denatures, e.g., casein precipitates as “cottage cheese”, but amino acids remained in clear solution. This “elemental diet” can be aspirated and re-fed without contributing to the risk of catheter blockage. We have used it exclusively since it became commercially available.

The harmless, but relatively distasteful and expensive formulation, was developed for the space program. Prisoner subjects subsisted exclusively for a year on this diet. The major effect was more complete absorption and reduced defecation to about once per week, which was NASA’s goal. We first reported another clinical indication for its use, production of a “physiological colostomy” in a hospitalized patient with otherwise refractory diverticulitis [15, 16].

## Methods

An 18 Fr (6 mm O.D.) duodenal feeding-decompression catheter is placed by either the nasal or gastrostomy route. The suction channel has gastric and post-pyloric aspiration orifices. The feeding channel terminates within the duodenum, just beyond the aspiration zone. Vivonex^®^ (Nestle HealthCare Nutrition, Inc.) is delivered immediately postoperatively by a feeding pump at the rate of 3000–5000 kcal (3000–5.000 ml) per day, Degassed aspirate is re-fed every 30 s by gravity flow.

Two 30 ml chambers are positioned five feet above the patient. Continuous 200 mm suction is applied alternately to these chambers at 30 s intervals. The level of suction actually experienced by the patients is reduced to approximately 50 mmHg by the height of the liquid column.

The aspirate is filtered, degassed, and directed by one-way valves to the chamber on suction. Simultaneously, aspirate collected the previous 30 s flows by gravity into the patients from the chamber off suction. The re-fed aspirate joins the continuous flow of elemental diet into the more distal duodenum via the feeding channel. The digestive enzymes in the recirculating aspirate cleanse the catheter’s aspiration and feeding channels.

### Ethical statement

All patients undergoing special studies or receiving experimental treatment had been reported previously, and the references cited. The assembled automatic control system described in this manuscript has not been commercialized, but each component has been approved for this clinical purpose by the US Food and Drug Administration for over a decade.

The rates of postoperative feeding are consistent with multiple clinical reports cited of the catheter feeding/decompression regimen alone, without the control system. Control system malfunction could only result in reduction of both the administered feedings and the re-fed aspirate.

### Consent statement

Written informed consent for participation in the study was obtained from each participant.

## Results and discussion

Some degree of paralytic ileus invariably follows major surgery. The impaired peristalsis with conventional enteral tube feeding techniques may incompletely propel the digestive juices, swallowed air, and the superimposed feedings. The excess would accumulate to distend at the duodenal feeding site, triggering the deleterious reflexes of “overfeeding”.

The patients’ endogenous contributions to duodenal inflow are approximately four liters per day, one liter each of saliva, bile, gastric and pancreatic juices. This inflow is less than 2.5 ml per 30 s cycle. Adding 3000–5000 ml of elemental diet per day raises total inflow to <6 ml per 30 s cycle.

With this regimen, the post-pyloric duodenum continuously is kept empty. Inflow is collected every 30 s. Swallowed air is vented, and liquid volumes greater than 30 ml overflow the collection chamber to be discarded permanently. The chamber’s residual content, ≤30 ml, is directed by the one-way valves to be re-fed into the more distal duodenum 30 s after collection.

This moderate inflow (<30 ml) under gravity into the distal duodenum over half a minute cannot disrupt function. If this volume is not totally propelled farther downstream, the excess will reflux into the aspiration zone, to be removed and discarded. However, peristalsis soon recovers sufficiently to propel the entire inflow. All the feedings and secretions are salvaged thereafter. In our experience, discarding of excess ceases within 2 h, and the daily total volume discarded averages only about 100–200 ml for the first postoperative day. It decreases thereafter as G-I function rapidly returns to fully normal.

A brief glance at the fluctuating levels in the collection chambers provides assurance that the monitor/control system is working properly. The impression that catheter occlusion is reduced and the nursing workload eased has not yet been objectively documented.

Our understanding of postoperative ileus and resultant under-nutrition very much parallels the earlier history of postoperative infection. Sepsis was accepted initially as normal, even inevitable, and many even believed that pus was beneficial, hence the archaic term, “laudable pus”. We now recognize that these are avoidable complications [17].

The primary culprit in postoperative ileus is the disruptive effect of swallowed air on the gut’s peristaltic pumping action. Immediately after surgery, we need to exclude air effectively from the G-I tract. This can be accomplished efficiently by simultaneous proximal duodenal aspiration, degassing, and more distal duodenal re-feeding. Priority needs to be given to all other efforts to minimize G-I dysfunction, e.g., early ambulation, avoidance of both pain and narcotics, etc. [18].

The duodenum and pylorus function as the natural “gatekeeper” for the gut. Even minimal duodenal distention is sensed, vagal reflexes close the pylorus, and all peristaltic activity slows.

Prompt restoration and exploitation of duodenal function are the keys to rapid recovery of the entire G-I tract.

The peristaltic gradient normally favors forward propulsion. Wangensteen conclusively showed experimentally that outflow from the distal duodenum would progress without further impediment immediately after bowel surgery, if swallowed air could be totally excluded from the gut’s “peristaltic pump”.

Intestinal absorption provides nutrients to the entire body by vascular distribution. However, the duodenum is “first at the trough” to meet its own nutritional needs. It can be nourished directly by intra-luminal elemental diet, supplementing nutrient delivery by the circulation. In the absence of distention, feedings both stimulate the duodenum to increase its peristaltic activity and fuel its ability to respond.

Current postoperative tube feeding regimens still follow “trial and error”. The initial rates are well below nutritional adequacy, and yet often exceed the patients’ reduced G-I function. This is recognized only after dysfunction worsens, with further slowing of peristalsis. First then is the feeding rate reduced, to be slowly increased thereafter, as tolerated. At best, this “conservative” approach prolongs suboptimal nutrition. Automating peristaltic monitoring and feeding control, supplemented by prevention of aerophagia, provide safe “enteral hyperalimentation” at a maximum pace, limited only by the patients’ own rates of G-I recovery.

The gut is a peristaltic pump whose effectiveness is always compromised by swallowed air. We ordinarily can (and must) tolerate a significant amount of intestinal gas and inefficiency.

However, this can readily deteriorate to an “air bound” state postoperatively. Total exclusion of swallowed air, in a sense, temporarily improves on nature. Protected peristaltic propulsion may even exceed preoperative basal levels.

With unprotected enteral catheter nutrition, excess foodstuff would accumulate at the feeding site in a pool of digestive enzymes, e.g., pancreatic amylase. Osmolality rises many-fold, and fluid is imbibed correspondingly. The initially minimal intestinal distention would increase because of this in situ digestion. Increasing distention is sensed by receptors within the intestinal wall, and the resulting vagal reflexes slow, halt, or even reverse the already impaired peristaltic activity.

The vascular component of these vagal reflexes probably triggers the rare, but usually fatal, bowel necrosis encountered in susceptible tube fed patients [19].

Controlled studies of conventional tube feeding following resective abdominal surgery were reported independently from Memorial Sloan-Kettering Cancer Center and the University of Ottawa [20, 21]. Postoperatively, all patients developed increased abdominal girth, but this manifestation of G-I dysfunction was more pronounced in the aggressively fed patients.

Unprotected tube feeding caused more greatly impaired respiratory function. These patients also had less spontaneous physical activity, especially ambulation, and had the sole incident of bowel necrosis. The authors of both studies concluded that early tube feeding by current techniques was too dangerous and unwarranted after major abdominal surgery.

To maximally yet safely exploit the potential benefits of postoperative enteral nourishment, we cannot rely on clinical observation alone to recognize and respond to the earliest stages of “overfeeding”. Inflow rates must be adjusted more rapidly to changes in peristaltic outflow than can be accomplished manually, to avoid even minimal and/or momentary local intestinal distention. Modern technology can be utilized to monitor peristaltic outflow relative to inflow automatically, and almost immediately prevent any excess feeding.

**Fig. 12:**
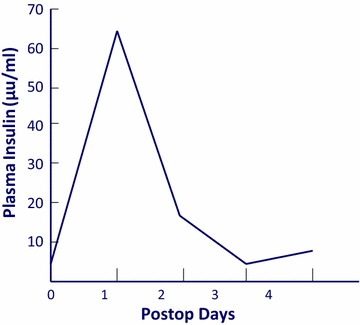
Postcholecystectomy insulin. Young, otherwise healthy “hyperalimented” females. Ref. [23]

Our patients under 30 years of age usually met the metabolic challenge of acute surgical stress plus a superimposed carbohydrate load. They achieved spontaneously elevated plasma insulin levels many-fold above basal [22] (Fig. 12). Diabetics and older patients required continuous blood glucose monitoring and insulin supplementation to maintain normoglycemia during the first postoperative day.

These patients were reassured that their postoperative need for supplemental insulin did not necessarily signify incipient diabetes. The effects of surgical stress on glucose metabolism abated within 24 h, following restoration of G-I function and absorption of normal oral nutrition.

When patients had “enteral hyperalimentation”, air was efficiently excluded, G-I activity was clinically normal by the morning following surgery. Patients had tolerated up to 5000 kcal of feedings during the initial 24 h following surgery, and were free of paralytic ileus symptoms. Uneventful discharge was achieved within 24 h for 160 consecutive “open” cholecystectomy patients, prior to the introduction of laparoscopy [23, 24]. We reported uneventful discharge within 24–48 h of “open” bowel resection [7, 25]. Bowel sounds were usually inaudible, despite the clinical and radiographic evidence of normal peristalsis, reflecting the almost complete absence of gas in the protected G-I tract.

## Conclusions

The postoperative effectiveness of the gut’s peristaltic pump recovers dramatically when swallowed air is totally excluded. Proximal duodenal aspiration, with monitoring and titration of the more distal duodenal outflow, permits safe absorption of about 5000 kcal/day of elemental diet immediately following major surgery. Almost all the digestive juices aspirated can be re-fed safely, with their contained secretory immune globulins, fluid, and electrolytes. Positive protein balance can be achieved by “enteral hyperalimentation” within hours of surgery, resulting in accelerated wound healing, enhanced immune competence, earlier discharge, etc. The returned digestive juices serve as an enzymatic cleansing flush of the catheter.

Symptomatic postoperative paralytic ileus is an avoidable complication.
